# Resectable carcinoma developing in the remnant pancreas 7 years and 10 months after distal pancreatectomy for invasive ductal carcinoma of the pancreas: report of a case

**DOI:** 10.1186/1477-7819-12-224

**Published:** 2014-07-18

**Authors:** Hiroya Akabori, Hisanori Shiomi, Shigeyuki Naka, Koichiro Murakami, Satoshi Murata, Mitsuaki Ishida, Yoshimasa Kurumi, Tohru Tani

**Affiliations:** 1 Department of Surgery,, Shiga University of Medical Science, Seta Tsukinowa-cho, Otsu, Shiga, Japan; 2 Division of Diagnostic Pathology, Shiga University of Medical Science, Seta Tsukinowa-cho, Otsu, Shiga, Japan; 3 Department of Surgery, National Hospital Organization Higashi-Ohmi General Medical Center, Gochi-cho, Higashi-Ohmi, Shiga, Japan

**Keywords:** Pancreatic carcinoma, Remnant pancreas, Repeated pancreatectomy

## Abstract

**Background:**

Pancreatic ductal adenocarcinoma, which represents 90% of pancreatic cancers, is one of the most lethal and aggressive malignancies. Operative resection remains the only treatment providing prolonged survival, however, recurrence of pancreatic ductal adenocarcinoma occurs in up to 80% of patients with pancreatic cancer within 2 years of a potential curative resection. There are few reports of pancreatic carcinoma recurrence (primary second cancer) in the remnant pancreas after pancreatectomy.

**Case presentation:**

A 52-year-old woman underwent a distal pancreatectomy for pancreatic cancer in September 2004. Adjuvant chemotherapy was started after surgery and continued for 4 years. In March 2012, marked elevation of DUPAN-II was observed, followed by an irregular stenotic finding in the main duct. We performed an *en bloc* resection of the remnant pancreas in July 2012. Histologically, the tumor contained a second primary pancreatic carcinoma with lymph node metastasis. At follow-up 20 months after the second operation, the patient was alive without recurrence. Fourteen cases of resectable cancer developing in the remnant pancreas after a pancreatectomy for cancer have been reported; a minority of these was identified as second primary tumors. Therefore, our patient’s primary second cancer is a rare event.

**Conclusion:**

The patient is considered to have shown a rare, unique pancreatic cancer recurrence. Persistent elevation of a tumor marker and extensive imaging led to proper diagnosis and treatment.

## Background

Pancreatic cancer remains one of the most difficult cancers to treat and has a very poor outcome. The American Cancer Society estimates that pancreatic cancer is the fourth leading cause of cancer mortality, with more than 40,000 new cases in 2012 [1]. The majority of patients initially present with clinically advanced disease, and only 10% to 15% are candidates for surgical resection. Despite the curative intent of surgical resection, cancer recurs within 2 years after pancreatic surgery in more than 60% of patients [2,3], and 5-year overall survival is only 12%, even with surgery [4]. The main problems facing surgeons treating pancreatic cancer are the frequency of late diagnosis, the lack of biomarkers that would allow early screening, and the absence of established radical strategies for treating recurrences. There have been several reports of pancreatic carcinoma recurring, or second primary lesions developing, in the remnant pancreas after pancreatectomy [5-16]. It is occasionally a critical issue to determine whether the tumor is a recurrence or is a second newly developed primary cancer. Wada *et al*. suggested molecular analysis and the clinicopathological findings are useful in discriminating between a recurrence versus a second primary cancer [6]. Here, we report a rare recurrence type of pancreatic cancer—a second developed primary carcinoma more than 7 years after the initial surgery—for which proper diagnosis and treatment resulted in a good outcome. We also review the associated literature.

## Case presentation

In August 2004, a 52-year-old woman was referred to our hospital because of an abnormal finding on abdominal ultrasonography (US) and computed tomography (CT) scan. Physical examination was unremarkable, and except for elevation of serum levels of DUPAN-II (1200 U/mL; normal range: 0–150 U/mL), all laboratory data were within the normal ranges. Abdominal US, CT scan, and magnetic resonance imaging (MRI) showed a small tumor in the body of the pancreas. Endoscopic retrograde cholangiopancreatography (ERCP) showed an obstruction (10 mm) in the main duct of the pancreatic body.

**Figure 1:**
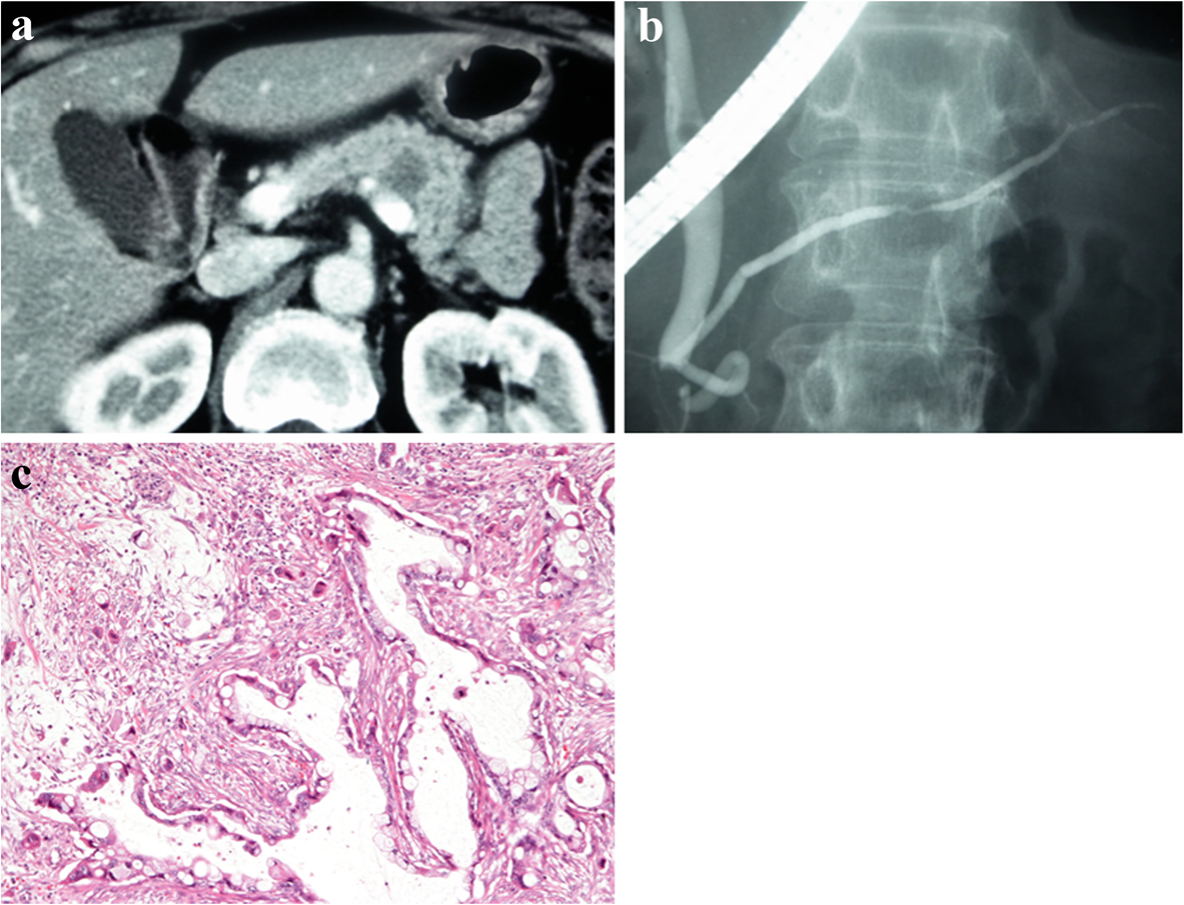
**Abdominal computed tomography (CT), endoscopic retrograde cholangiopancreatography (ERCP), and histological findings of the first pancreatic cancer. (a)** Abdominal CT showed a low-density mass in the body of the pancreas. **(b)** ERCP demonstratedan obstruction of the main pancreatic duct in the pancreatic body. **(c)** Histological examination of the resected specimen at the first operation showed a moderately to poorly differentiated tubular adenocarcinoma. Hematoxylin and eosin, magnification × 100.

With a preoperative diagnosis of ductal carcinoma of the pancreas, distal pancreatectomy and peripancreatic lymph node dissection were performed on September 2004. There were no abnormal findings in the head of the pancreas during surgery, and the cut end of the pancreas was judged to be negative for cancer, based on intraoperative histological examination of frozen sections. The histological diagnosis was a moderately to poorly differentiated tubular adenocarcinoma with perineural invasion, lymphatic and venous permeation, and retroperitoneal invasion (pStage IIA, pT3pN0M0 according to the International Union against Cancer (UICC) TNM classification [17]) (Figure 1).

The surgical margins of the resected specimen were free of atypical or cancerous cells. The postoperative course was uneventful, and the serum level of DUPAN-II declined to a normal level. Even though systemic adjuvant chemotherapy was performed with gemcitabine hydrochloride (1000 mg/m^2^ body weight once a week for 2 weeks, followed by 1 week of rest; constituting one treatment cycle) from October 2004 to November 2007, the serum DUPAN-II level gradually rose to 400 U/mL after January 2007. Therefore, a recurrence of the pancreatic cancer was suspected, but there was no evidence of recurrence using several imaging techniques.

In January 2008, a thoracoscopic right upper lobectomy was performed under the diagnosis of a primary lung cancer of the right upper lobe. The histological diagnosis was a primary adenocarcinoma of the lung, combined type (mucinous BAC + papillary type; pStage IA, pT1pN0M0 according to the UICC TNM classification). Six years after the initial pancreatic operation, the DUPAN-II level had risen to 800 U/mL in the absence of clinical signs of recurrence. In March 2012, endoscopic ultrasonography and ERCP confirmed pancreatic cancer recurrence with an irregular stenotic finding in the main duct of the pancreatic head and a dilated distal main duct, associated with marked elevation of the DUPAN-II level to more than 1,400 U/mL. Cytology of the pancreatic juice was negative for cancer cells. Fluorodeoxyglucose positron emission tomography (FDG-PET) reconfirmed the absence of any distant metastasis or mass lesions in the remnant pancreas.

**Figure 2:**
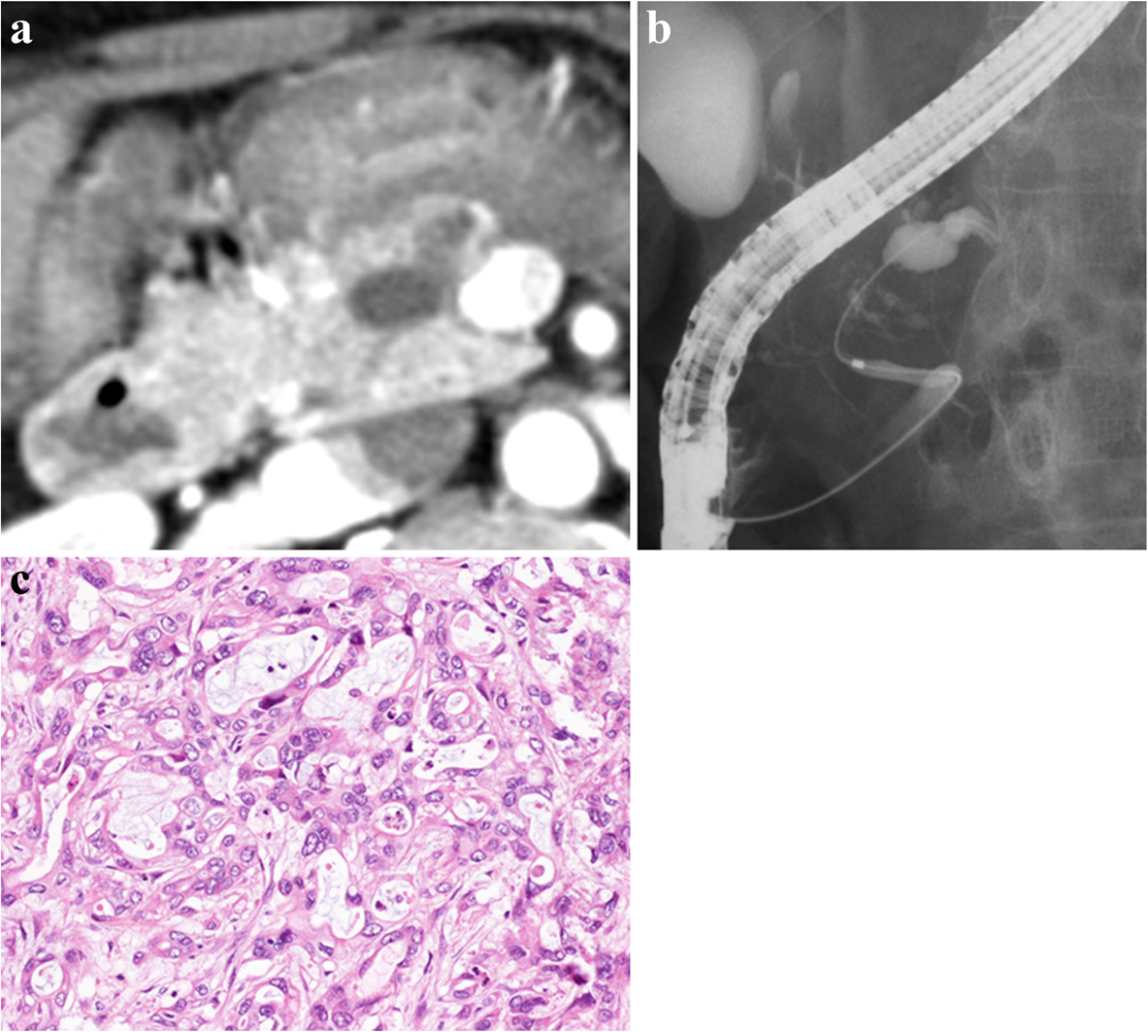
**Abdominal computed tomography (CT), endoscopic retrograde cholangiopancreatography (ERCP), and histological findings of the second pancreatic cancer. (a)** Follow-up CT revealed a dilated distal pancreatic duct with no mass in the remnant pancreas. **(b)** Follow-up ERP demonstrateed a stricture of the main pancreatic duct in the head of the pancreas and a markedly dilated distal pancreatic duct. **(c)** Histological examination of the remnant pancreatic tumor showed a well to moderately differentiated tubular adenocarcinoma. Hematoxylin and eosin, magnification × 400.

Because the patient was in good clinical condition, a second pancreatic operation was performed in July 2012. The remnant pancreas and peripancreatic lymph nodes were resected, and hepaticojejunostomy and gastrojejunostomy were performed. Histologically, the second pancreatic neoplasm was diagnosed as an invasive ductal carcinoma composed of a well to moderately differentiated tubular adenocarcinoma with no infiltration into the microvessels (Figure 2). *In situ* carcinoma was detected in the main pancreatic duct around the invasive lesion. There was no invasion into the retropancreatic soft tissue, and the surgical margins of the resected specimen were free of atypical or cancerous cells. Regional lymph node metastasis was found and was classified as pStage IIB (pT1pN1pM0) and R0. At follow-up 9.5 years after the first operation and 20 months after the second operation, the patient was alive without cancer recurrence and the DUPAN-II concentration had normalized to 140 U/mL.

## Discussion

In this case, recurrent carcinoma in the remnant pancreas was successfully removed 7 years and 10 months after distal pancreatectomy for invasive ductal carcinoma in the body of the pancreas.

In the literature, we found 14 cases of a resectable cancer developing in the remnant pancreas after pancreatectomy for cancer of the pancreas (Table 1). When cancer is detected in the remnant organ after resection of a tumor with curative intent, it can be difficult to determine whether it is a recurrence of the first cancer or a second, newly developed, primary cancer. In the 14 cases we identified in the literature (Table 1), 9 were diagnosed as a recurrence and 5 as a second primary tumor in the remnant pancreas (1 report did not contain information about the origin of the second tumor).

**Table 1:** Summary of resectable carcinoma developing in the Remnant pancreas after pancreatectomy for pancreatic cancer

**No.**	**Author**	**Year**	**Age/sex**	**Time to recurrence, months**	**Operation (first/second)**	**Stage (first/second)**	**Adjuvant therapy**	**TM elevation**	**Mass detected by imaging**	**Site of recurrence at second operation**	**Survival after second operation, months**
1	Eriguchi *et al*. [5]	2000	67/F	88	DP/TP	IA/IIB	No	CA19-9	+	Primary	8^a^
2	Wada *et al*. [6]	2001	52/F	22	PPPD/TP	IIB/-	CT	CA19-9	+	Local	44
3	D’Amato *et al*. [7]	2002	44/M	40	PPPD/TP	IIB/-	CRT	CA19-9	+	NR	22^a^
4	Doi *et al*. [8]	2003	60/M	26	DP/TP	IIA/IB	No	No	+	Primary	7
5	Takamatsu *et al*. [9]	2005	63/M	43	PD/TP	IIA/IIA	CT	CA19-9	+	Primary	10^a^
6	Dalla *et al*. [10]	2006	63/M	12	PD/TP	IIA/IIA	CRT	CA19-9	+	Local	24^a^
7	Miura *et al*. [11]	2007	72/F	29	PPPD/DP	IIA/IV	NR	NR	+	Local	5
8	Tajima *et al*. [12]	2008	58/M	34	PPPD/TP	IB/IB	No	CA19-9	+	Local	38^a^
9	Koizumi *et al*. [13]	2010	65/M	85	PPPD/TP	IA/IIB	CT	No	+	Local	10^a^
10		2010	67/M	25	DP/TP	IA/IIB	No	CA19-9	+	Local	8^a^
11	Ogino *et al*. [14]	2010	63/F	71	PPPD/TP	IIB/IB	CT	CA19-9	+	Local	13^a^
12		2010	56/M	37	PPPD/TP	IIB/IA	CT	CA19-9	+	Local	7^a^
13	Kinoshita *et al*. [15]	2012	58/F	68	PD/TP	IIB/-	CT	CEA, CA19-9	+	Primary	2^a^
14	Kobayashi *et al*. [16]	2012	58/F	38	PPPD/TP	IIB/IA	CT	CA19-9	+	Local	20^a^
15	Present case	2013	52/F	94	DP/TP	IIA/IIB	CT	DUPAN-II	-	Primary	20^a^

CRT, chemoradiotherapy; CT, chemotherapy; DP, distal pancreatectoomy; NR, not reported; PD, pancreaticoduodenectomy; PPPD, pylorus-preserving pancreaticoduodenectomy; TM, tumor marker; TP, total pancreatectomy.

^a^Alive at last follow-up.

In our patient, we could not analyze the tumor for mutations in the *ras* gene, the most prevalent type of mutation present in pancreatic cancer [18], because the specimen from the initial surgery was not stored, but we judged the second neoplasm to be a second primary tumor for the following reasons. 1) The histopathological diagnosis of the first tumor was moderately to poorly differentiated tubular adenocarcinoma and the surgical margins of the resected specimen were free of atypical cells, and were sufficiently distant from the tumor. By contrast, the second pancreatic carcinoma was a well to moderately differentiated tubular adenocarcinoma, and *in situ* carcinoma was detected in the main pancreatic duct around the invasive lesion. 2) The fact that this patient had lived for 7 years and 10 months after the resection (the longest interval among the reported cases) is also evidence that this was likely to have been a second primary tumor. However, we cannot completely rule out that this was a recurrence of the original cancer and cannot definitively prove the tumor etiology. In our patient, long-term careful follow-up with several imaging and tumor markers provided valuable information for the diagnosis of remnant pancreatic carcinoma.

The typical findings that lead to the detection of pancreatic neoplasms include a mass lesion on CT and MRI, as well as an irregular stricture in the main pancreatic duct and dilation of the distal pancreatic duct, seen with ERCP and associated with the elevation of tumor marker levels. Koizumi [13] reported that FDG-PET can provide valuable information for the diagnosis of remnant pancreatic carcinoma, even when CT or MRI shows no obvious tumor. In the previous reports (Table 1), imaging detected mass lesions in 14 cases (93%), and 12 (86%) had elevation of the tumor marker CA19-9 (1 report had no information on these measures). However, our patient was unusual because several imaging evaluations, including FDG-PET, did not detect a mass lesion in the remnant pancreas before the second operation (Figure 2), which made it difficult for us to definitively diagnose recurrent pancreatic carcinoma. In this case, the plasma CA19-9 levels had remained within the normal limits (data not shown), however, the elevated serum DUPAN-II level strongly indicated a potential malignancy, and caused us to carry out appropriate follow-up imaging examinations. When a serum tumor marker is elevated during the follow-up period, several periodic imaging evaluations (US, CT, FDG-PET, and ERCP) are necessary and useful investigative techniques for early detection of recurrence in the remnant pancreas.

## Conclusion

Our patient developed pancreatic cancer in the remnant pancreas 7 years and 10 months after distal pancreatectomy. The persistent elevation of a serum tumor marker, accompanied by detailed imaging examinations, led us to make the correct diagnosis and give appropriate treatment with good results. Further study is needed to determine whether pancreatectomy for a recurrent tumor that is limited to the remnant pancreas improves patient prognosis.

## Consent

Written informed consent was obtained from the patient for publication of this case report and any accompanying images. A copy of the written consent is available for review by the Editor-in-chief of this journal.

## Abbreviations

CT: Computed tomography; ERCP: Endoscopic retrograde cholangiopancreatography; FDG: Fluorodeoxyglucose; MRI: Magnetic resonance imaging; PET: positron emission tomography; UICC: Union for International Cancer Control; US: Ultrasonography.

## Competing interests

The authors declare that they have no competing interests.

## Authors’ contributions

HA, SN, and TT acquired the data and wrote the manuscript. HA, HS, KM, SM, and YK performed the surgery. MI was the leading pathologist, and provided the histological examinations. SM performed the clinical follow-up of the patient. All authors read and approved the final manuscript.
